# In vitro metal leaching from tampons under simulated vaginal and menstrual pH conditions

**DOI:** 10.1016/j.xagr.2026.100686

**Published:** 2026-08-25

**Authors:** Gillian S. Kaneshiro, Daysi Martinez, Heidi Hannoush, Marianthi Anna Kioumourtzoglou, Jenni A. Shearston, Nancy Reame, Kristen Upson, Kathrin Schilling

**Affiliations:** 1 Department of Environmental Health Sciences, Mailman School of Public Health, Columbia University, New York, NY (Kaneshiro, Martinez, Hannoush, Kioumourtzoglou, Schilling); 2 Department of Epidemiology, School of Public Health, Brown University, Providence, RI (Kioumourtzoglou); 3 Institute at Brown for the Environment and Society, Brown University, Providence, RI (Kioumourtzoglou); 4 Department of Integrative Physiology, University of Colorado Boulder, Boulder, CO (Shearston); 5 Columbia University School of Nursing, New York, NY (Reame); 6 Department of Epidemiology and Biostatistics, College of Human Medicine, Michigan State University, East Lansing, MI (Upson)

**Keywords:** environmental exposure, leaching, menstruation, metals, tampons

## Abstract

**BACKGROUND:**

Approximately 100 million people worldwide use tampons to manage their menstruation. Tampons have been shown to contain toxic metals, which may contribute to cumulative exposure risk. It is unclear whether metals leach from tampons or how changes in vaginal pH during menstruation affect leaching.

**OBJECTIVE:**

To quantify in vitro metal leaching from tampons under physiologically relevant conditions.

**METHODS:**

We assessed metal leaching from 22 tampon products by saturating them in simulated vaginal (pH 4.2) and menstrual fluids (pH 7.4) for 8 hours at 37°C on a shaker. Extracted supernatants were analyzed for 15 metals by inductively coupled plasma triple quadrupole mass spectrometry. Metal leaching was compared across 13 brands and tampon characteristics: organic composition and absorbency. The leaching fraction—percentage of total metal content leached—was calculated using data reported by Shearston et al.

**RESULTS:**

We found measurable amounts of 14 metals in leachates from vaginal fluid and 12 metals in menstrual fluid, including lead, arsenic, and cadmium. Most metals leached in greater amounts in vaginal fluid compared to menstrual fluid, such as lead (mean: 40.3 ng, 95% confidence interval around mean value: [6.94, 73.7] vs 15.6 ng, [6.91, 24.3]). The highest average leaching fraction in vaginal fluid was copper (233%, [95% CI: −87% to 553%]), and in menstrual fluid, nickel (53%, 95% CI: 38%–79%]). Metal leaching varied by tampon characteristics.

**DISCUSSION:**

Under simulated physiological conditions, we found that tampons leach metals in both vaginal fluid and menstrual fluid. Since in vitro metal release does not equate to vaginal absorption or systemic exposure, further investigations are required to inform the risk of metal exposure from tampon use.

**AJOG Global Reports at a Glance**

**Why was this study conducted?**

Metals have been detected in tampons, but it remains unclear whether they leach under physiologically relevant vaginal and menstrual conditions and contribute to exposure.

**Key findings**

Tampons released multiple metals, including lead, arsenic, and cadmium, with higher amounts in vaginal fluid than menstrual fluid. Metal release varied by tampon characteristics, and a measurable fraction of total metal content was released.

**What does this add to what is known?**

Provides quantitative evidence of metal leaching under realistic conditions and introduces leaching fraction as a metric to assess potential exposure.

## Introduction

Nearly half of the world’s population menstruates. More than 100 million people in more than 120 countries use tampons for at least part of their menstrual protection.^1^ In the United States, up to 86% of menstruating individuals, including 71% of adolescents, use tampons.^1^ Tampons are designed to be worn in the vagina, where they absorb menstrual fluid for up to a recommended duration of 8 hours.^2^ Over a typical reproductive lifespan, from the average age of menarche at age 12 to menopause at age 52, a menstruator may use approximately 10,000 tampons,^3^, ^4^, ^5^ highlighting the potential for cumulative chemical exposure.

Despite their widespread use, tampons are not subject to ingredient disclosure requirements in a majority of the United States.^6^^,^^7^ FDA’s testing recommendations focus on biocompatibility and preclinical microbiology, but not comprehensive chemical testing.^7^ Consequently, consumers may be unaware of chemical additives or contaminants. Toxic chemicals have been detected in menstrual products, including dioxins, phthalates, parabens, per- and polyfluoroalkyl substances (PFAS), and volatile organic compounds.^8^^,^^9^ One study found that a tampon brand leached heliotropin, an allergenic fragrance, in amounts that could trigger allergic contact dermatitis in sensitized individuals.^10^ Metals and other small molecules may be bioavailable depending on their chemical form and product characteristics. However, the extent to which this applies to metals remains uncertain. Previous studies (reviewed by Upson et al^11^) have reached varying conclusions about tampon-related chemical exposures, and some have relied on dermal absorption data, which do not fully reflect vaginal physiology.

Studying metal leaching from tampons is critical given the unique features of the vagina, which allow for efficient absorption.^12^^,^^13^ The vaginal epithelium is uniquely susceptible to chemical uptake due to its high permeability and large mucosal surface area.^14^ Chemicals absorbed by the vagina bypass hepatic first-pass metabolism and enter the bloodstream directly.^15^ Drug delivery studies have shown significantly higher bioavailability for vaginal vs oral administration of compounds such as misoprostol and estradiol.^12^^,^^16^^,^^17^ Estradiol, for example, reached 10 to 80 times higher concentration after vaginal administration compared to oral intake.^18^, ^19^, ^20^ Vaginal pH may further contribute to chemical leaching under the influence of menstrual cycle dynamics.^21^ Early in the menstrual cycle, after the onset of menses, menstrual fluid—which is naturally alkaline—raises the vaginal pH, effectively making it neutral. As menstruation progresses and flow decreases, cervical and vaginal secretions (which have a lower pH) predominate, restoring an acidic vaginal environment.^22^, ^23^, ^24^

Data for metal content of menstrual products are sparse, and it is unclear whether metals leach from tampons. A prior study investigating the chemical composition and leaching of menstrual and intimate care products did not find significant levels of metals in tampons.^25^ In contrast, our previous study identified measurable concentrations of 16 metals in tampons; 12 metals were detected in all tested tampons.^5^ Several of these metals are known metalloestrogens (eg, arsenic [As], cadmium [Cd], copper [Cu], lead [Pb], mercury [Hg], nickel [Ni], and zinc [Zn]), capable of mimicking the action of endogenous estrogens and disrupting hormonal signaling.^26^^,^^27^ Pb, which has no safe level of exposure, has been associated with reduced fertility, miscarriage, and premature birth.^28^^,^^29^ Furthermore, Pb and Cd have biological half-lives of 10 to 30 years and accumulate in bones and soft tissues, displacing essential elements like calcium (Ca) and iron (Fe).^27^^,^^30^^,^^31^

To better define the contribution of tampons to human metal exposures, we evaluated the leaching of 15 metals (As, barium [Ba], Cd, cobalt [Co], chromium [Cr], Cu, Fe, Hg, manganese [Mn], Ni, Pb, selenium [Se], strontium [Sr], vanadium [V], and Zn) from tampons under simulated menstrual conditions. We determined metal leaching (total and percent) from the same tampon products previously analyzed for total metal content by Shearston et al,^5^ including a range of brands, absorbencies, and both organic and nonorganic products, using simulated vaginal fluid (pH 4.2) and menstrual fluid (pH 7.4).

## Materials and methods

### Tampon sample selection

For the leaching experiments, most of the products had previously been assessed for total metal content and reported by Shearston et al.^5^ The tampons were purchased between September 2022 and March 2023 from retail stores in the United States (New York City), the European Union (only two products), as well as from two US-based major online retailers. A summary of tampon characteristics (with brand and product line names undisclosed) is provided in Table 1.

**Table:** Characteristics of tampon products included in the leaching study.

Brand	Product line	Organic composition	Place of purchase	Absorbency	Number of tampons	Number of samples	Fluid tested
A	1	Nonorganic	US	Regular	2	4	Vaginal, Menstrual
A	3	Nonorganic	US	Regular	2	3	Vaginal, Menstrual
A	3	Nonorganic	US	Super	1	1	Menstrual
B	4	Nonorganic	US	Super	1	1	Menstrual
C	5	Nonorganic	US	Regular	2	3	Vaginal, Menstrual
C	5	Nonorganic	US	Super	2	3	Vaginal, Menstrual
D	7	Organic	US	Super	1	1	Vaginal
E	8	Nonorganic	US	Regular	1	1	Menstrual
E	8	Nonorganic	US	Super	2	3	Vaginal, Menstrual
F	9	Organic	US	Regular	2	2	Vaginal, Menstrual
F	9	Organic	US	Super	2	2	Vaginal, Menstrual
G	10	Organic	US	Regular	2	4	Vaginal, Menstrual
G	10	Organic	US	Super	1	1	Menstrual
I	12	Nonorganic	US	Regular	2	3	Vaginal, Menstrual
I	12	Nonorganic	US	Super	2	3	Vaginal, Menstrual
J	14	Nonorganic	US	Regular	2	2	Vaginal, Menstrual
J	14	Nonorganic	US	Super	2	2	Vaginal, Menstrual
K	15	Nonorganic	US	Regular	2	2	Vaginal, Menstrual
K	15	Nonorganic	US	Super	1	1	Vaginal
L	16	Nonorganic	EU/UK	Regular	1	1	Vaginal
M	17	Organic	EU/UK	Regular	1	1	Vaginal
N	18	Organic	US	Super	2	4	Vaginal, Menstrual

Decoding of brand and product line for products based on Shearston et al.^5^

### Leaching experiment

Prior to the experiment, the background metal concentrations present in both simulated vaginal fluid and simulated menstrual fluid were measured (Supplemental Table 1). These baseline concentrations were subtracted from the measured values in the leaching experiments. The saturation volumes of the two tampon sizes (regular and super) were determined using nontest tampons of the same product lines. Each tampon was submerged in deionized water at room temperature until fully hydrated, indicated by a thin surface film of water. Saturation volume was calculated from the mass difference between the dry and saturated tampon (assuming 1 g ≈ 1 mL). Based on replicate measurements, the saturation threshold was 10.5 mL for regular absorbency and 16.0 mL for super absorbency. This ensured full absorption, enabling efficient fluid retrieval and retention analysis.

To avoid variations in composition, pH, and viscosity of vaginal secretions within and between individuals, leaching experiments used commercially-prepared, simulated vaginal (BZ181) (pH 4.2) and menstrual (BZ266) (pH 7.4) fluids obtained from Biochemazone (Leduc, Canada).^32^ BZ181 replicates natural vaginal secretions and includes mineral salts (Na⁺, K⁺, Ca²⁺, Mg²⁺, chloride, phosphate, sulphates), stabilizers, metabolites (lactic acid, urea, creatinine, uric acid), sugars, lysozyme, amylase, lipids, glycogen, amino acids, vitamins, serum proteins, and mucin, but lacks blood cells, hemoglobin, epithelial cells, and immunoglobulins. BZ266 contains buffered salts, serum proteins (including albumin), metabolites, lipids, glycogen, amino acids, glucose, lysozyme, mucin, amylase, viscosity enhancers, and hemoglobin.

Tampons were unpacked, applicators removed (if present), and placed in 50 mL metal-free centrifuge tubes. Most tampons (*n*=14) were tested in both simulated vaginal and menstrual fluids, but due to product depletion from a prior study,^5^ 8 of the 22 products were tested in only one fluid type (vaginal *n*=4; menstrual *n*=4) (Table 1). In total, 12 brands were tested with vaginal fluid and 10 brands with menstrual fluid. To assess reproducibility, 12 product–fluid combinations were tested in duplicate. Tampons were incubated at 37°C for 8 hours—the FDA-recommended maximum use duration—on a vertical shaker to simulate body movement.^33^ Following incubation, fluids were extracted under a laminar flow hood using Teflon-coated tweezers. Each sample yielded 1 to 2 mL of leachate for metal analysis.

### Metal analysis

The analysis was performed at Columbia University’s Trace Metal Core Facility using inductively coupled plasma triple quadrupole mass spectrometry (ICP-QQQ-MS, Agilent 8900). Detailed information about instrumental settings is provided in the Supplemental Information. The limit of detection (LoD) was defined as 3.33 times the standard deviation of simulated fluid blank measurements (Supplemental Table 1 Panel C). The method detection limit (MDL) was calculated by multiplying the LoD by a dilution factor of 10.5 and 16.0 for regular and super-absorbency tampons, respectively, and reported in nanograms. The values for MDL are reported in Supplemental Tables 2 and 3.

### Statistical analysis

We calculated the mean leached mass and corresponding 95% confidence intervals (CIs) for each metal across all tampons tested in simulated vaginal fluid and simulated menstrual fluid. Relative standard deviations for metals measured in duplicate tampon leaching experiments are reported in Supplemental Table 4. Next, we compared the average amount of metals leached by tampon product characteristics, including (1) brand (grouping different product lines of the same brand together), (2) organic and nonorganic tampons, (3) tampon absorbency (regular and super). We did not conduct formal statistical significance testing between comparisons due to small group sizes (Table 1). Lastly, we calculated the leaching fraction—the proportion of each metal that is bioaccessible under realistic use conditions—as the percentage of metal leached using the total metal content reported by Shearston et al^5^ (Supplemental Table 5 Panel A). Additional tampons were tested for metal analysis using the same method as described in Shearston et al,^5^ and the total metal content is reported in Supplemental Table 6. Statistical analyses were performed in R version 4.4.2 using the *tidyverse* package.^34^

## Results

### Metals leached from tampons

We analyzed 22 commercially available tampons products across brands and product lines to quantify leachable amounts of 15 metals. Eighteen were tested in simulated vaginal fluid and 18 in simulated menstrual fluid (Table 1). All 15 metals were detectable in vaginal fluid leachates (Supplemental Table 2), whereas only 12 were detected in menstrual fluid (Supplemental Table 3). After correcting for background levels, Fe, Hg, and Se were below the MDL in menstrual fluid. Leachable amounts varied widely by metal (Figure 1). Zn showed the highest levels under both conditions, ranging from 351 to 107,124 ng in vaginal fluid and 592 to 39,492 ng in menstrual fluid (Supplemental Tables 2 and 3). Cd and V had the lowest detectable masses, averaging 4.99 ng (95% CI for the mean: 2.51, 7.48) and 4.50 ng (95% CI: −0.283, 9.28) in vaginal fluid, and 4.64 ng (95% CI: 2.47, 6.82) and 7.51 ng (95% CI: −0.185, 15.2) in simulated menstrual fluid, respectively. As and Cr also leached in relatively small amounts. Mean leached amounts were higher in vaginal fluid (pH 4.2) than in menstrual fluid (pH 7.4): 8.47 ng (95% CI: 4.13–12.6) versus 6.31 ng (95% CI: 2.80–9.82) for As, and 13.6 ng (95% CI: 9.58–17.7) versus 10.3 ng (95% CI: 8.29–12.4) for Cr (Figure S1). In contrast, Ni leached in greater amounts in menstrual fluid..

**Figure 1:**
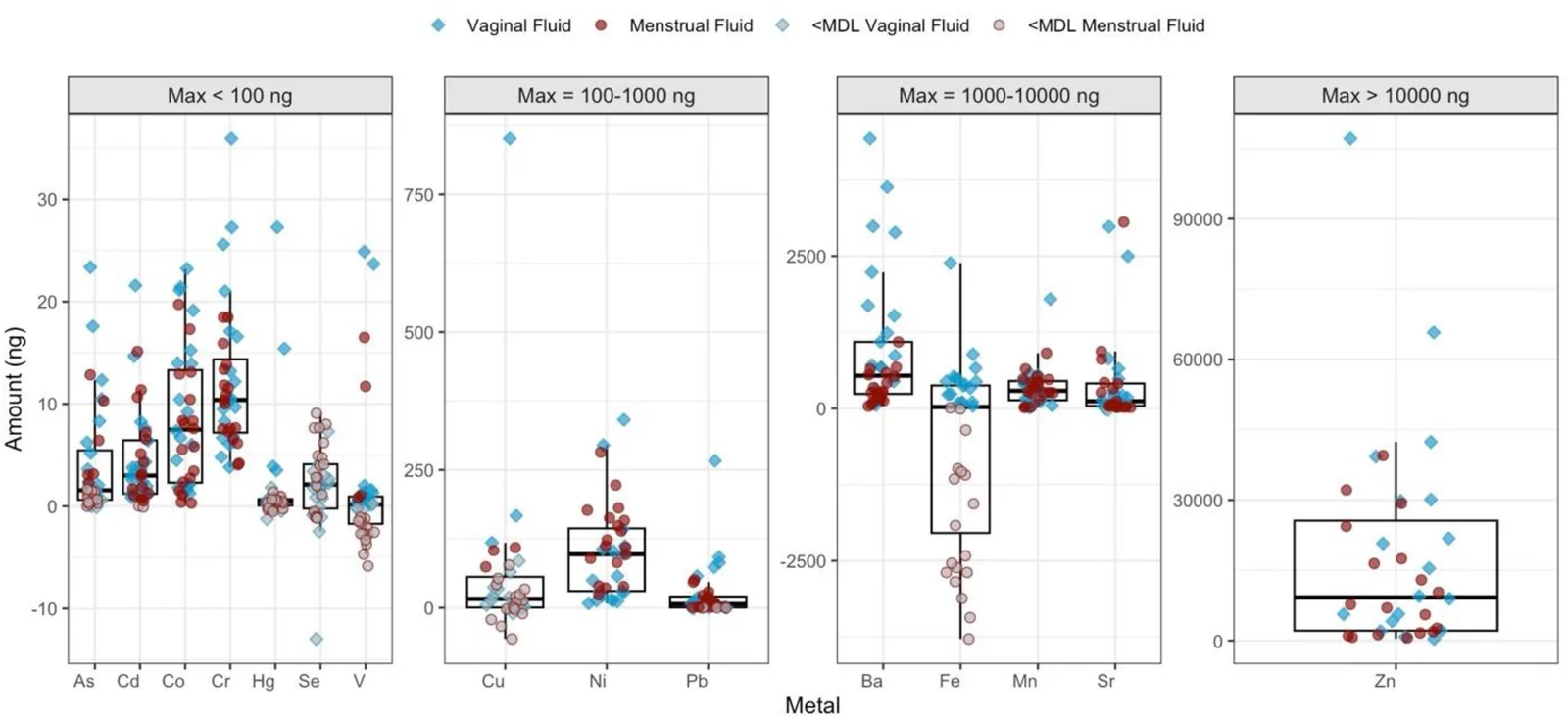
Metal leachate levels (ng) from tampons exposed to simulated vaginal fluid (blue diamond, pH 4.2) and menstrual fluid (red circle, pH 7.4) Values below MDL for each metal are shown in grey and outlined in blue or red to correspond to the respective simulant fluid. The leachate levels are grouped by maximum amount of metal leached <100 ng, 100 to 1000 ng, 1000 to 10,000 ng, and >10,000 ng.

Kaneshiro. In vitro metal leaching from tampons under simulated vaginal and menstrual pH conditions. AJOG Glob Rep 2026.

Leaching fractions exceeding 100% of the total metal content are possible and do not indicate a mass balance issue. Leaching fractions were calculated relative to total tampon metal concentrations reported by Shearston et al.^5^. As total metal content and leached metal mass were measured on separate tampons from the same product line, unit-to-unit variability in metal content can yield differences in leaching fractions above 100% relative to total metal measured in a different tampon. These values should therefore be interpreted as reflecting variability in total metal concentrations across individual products and studies rather than complete release of the metal from a single tampon.

### Variability of metal leaching by tampon characteristics

We observed a large variability in the average amount of metal leached across tampon brands. Metal leachate levels varied more than 10-fold across brands for certain elements such as Pb and Zn in both simulated fluid conditions (Figure 2). In simulated vaginal fluid, some metals, such as As, Cu, and V, were not detectable across all brands, whereas other metals, such as Ba, Co, and Mn, leached from all tested brands (Figure 2, A). Similar variability for these metals was observed in simulated menstrual fluid (Figure 2, B).

**Figure 2:**
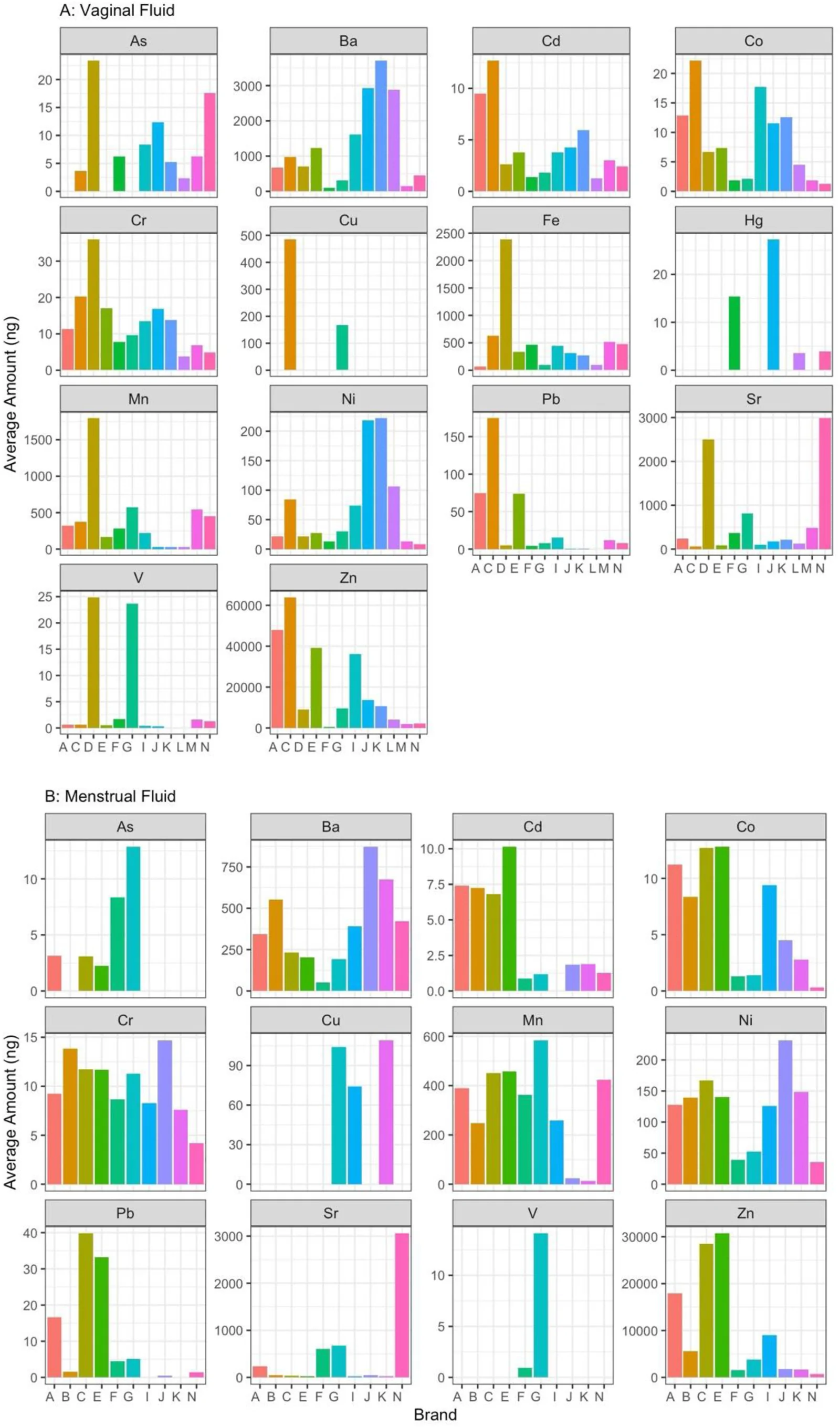
Metal leachate levels (ng) from tampons across 12 different brands exposed to (A) simulated vaginal fluid (pH 4.2) and 10 brands in (B) menstrual fluid (pH 7.4) Values below the method detection limit (MDL) were excluded from the analysis.

Kaneshiro. In vitro metal leaching from tampons under simulated vaginal and menstrual pH conditions. AJOG Glob Rep 2026.

Differences between the amounts of metal leached from organic vs nonorganic tampons were relatively consistent when tested with simulated vaginal and menstrual fluid (Figure 3). Under both conditions, Cd, Co, Ni, Pb, and Zn leached in greater quantities from nonorganic tampons compared to organic tampons. On the other hand, As, Sr, and V leached at higher amounts from organic tampons compared to nonorganic tampons. We also found differences in the average amount of metal leached when comparing tampon absorbency (Figure 4). All regular-absorbency products leached less metal per tampon than super absorbency product for all detectable metals when saturated in both simulated vaginal and menstrual fluid.

**Figure 3:**
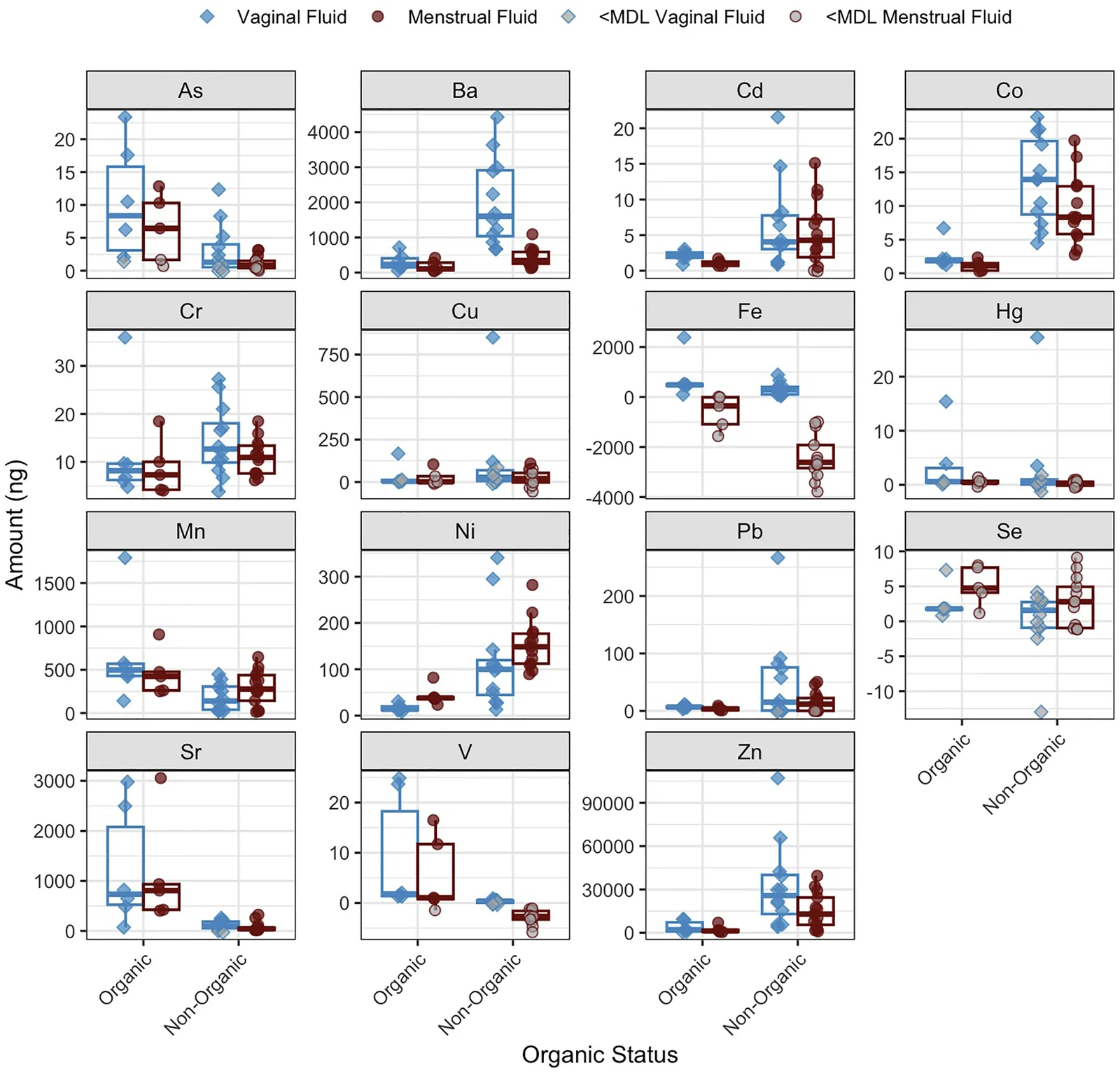
Metal leachate levels (ng) from organic and nonorganic tampons exposed to simulated vaginal fluid (blue diamond, pH 4.2) and menstrual fluid (red circle, pH 7.4) Values below MDL for each metal are shown in grey and outlined in blue or red to correspond to the respective simulant fluid.

Kaneshiro. In vitro metal leaching from tampons under simulated vaginal and menstrual pH conditions. AJOG Glob Rep 2026.

**Figure 4:**
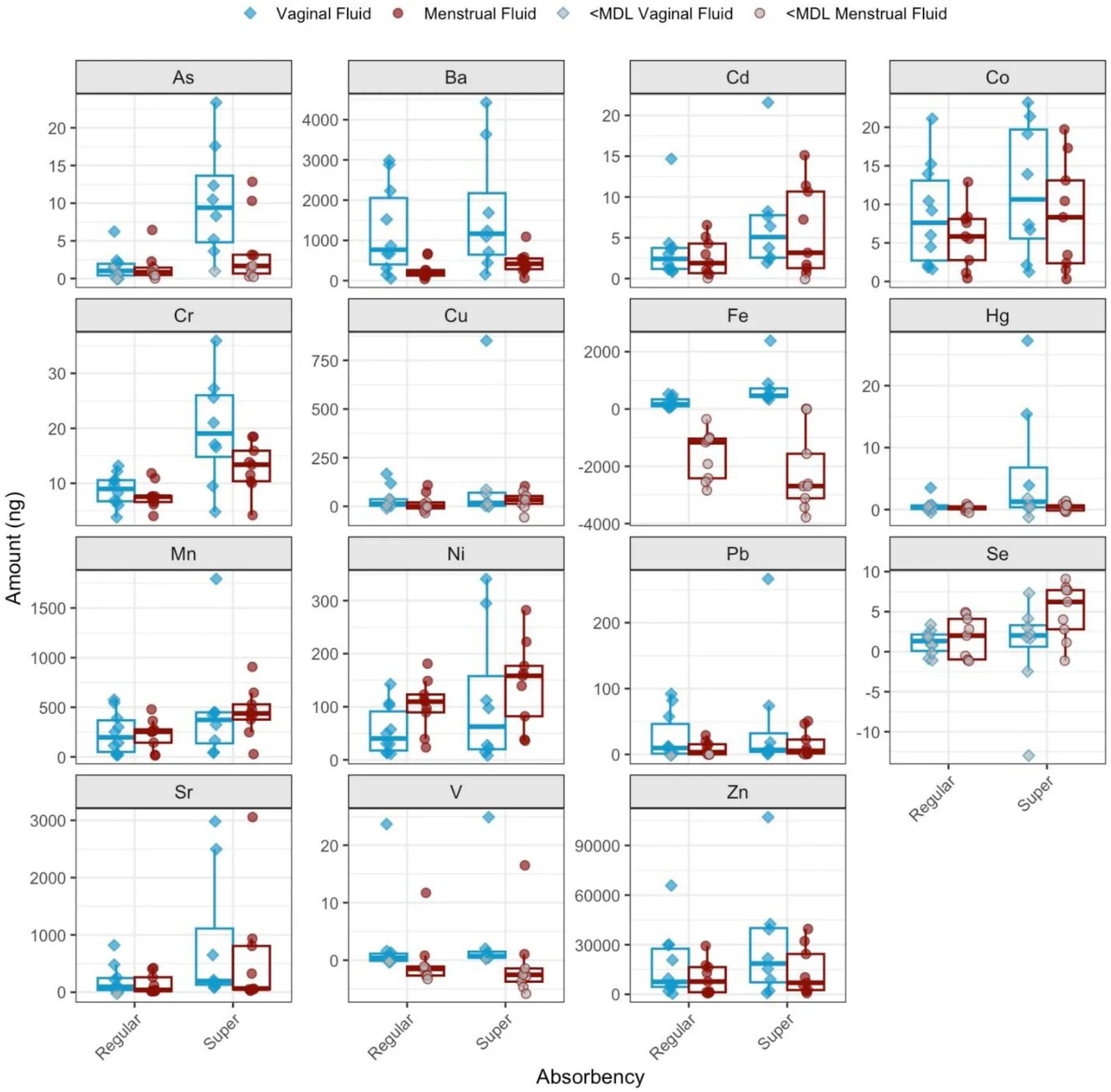
Metal leachate levels (ng) from regular and super-absorbency tampons exposed to simulated vaginal fluid (blue diamond, pH 4.2) and menstrual fluid (red circle, pH 7.4) Values below MDL for each metal are shown in grey and outlined in blue or red to correspond to the respective simulant fluid.

Kaneshiro. In vitro metal leaching from tampons under simulated vaginal and menstrual pH conditions. AJOG Glob Rep 2026.

### Tampon leaching fraction

We define the leaching fraction as the ratio of leached metal mass to total metal content in the product. The average metal leaching fraction was calculated for 13 of 15 metals and was higher in simulated vaginal fluid compared to simulated menstrual fluid for 10 metals (Supplemental Table 5, Panel B (B1 for vaginal, B2 for menstrual)). This included As (128% [95% CI: 46%– 210%] vs 48% [95% CI: 31%–65%]), Cd (38% [95% CI: 28%–49%] vs 24% [95% CI: 18%–30%]), and Pb (12% [95% CI: 5%–19%] vs 5% [95% CI: 2%–7%]). Only Ni had a higher average leaching fraction in simulated menstrual fluid (58% [95% CI: 38%–79%]) compared to simulated vaginal fluid (26% [95% CI: 15%–37%]). Cr, Mn, and V leached similarly in both fluids (5% [95% CI not calculated], 35% [95% CI: 28%–41%], and 7% [95% CI: 3%–11%] in simulated vaginal fluid, respectively, compared to 5% [95% CI: 5%–6%], 38% [95% CI: 31%–44%] and 6% [95% CI: 0.1%–11%] in simulated menstrual fluid.

The leaching fraction for tampons varied widely across brands and metals (Supplemental Table 5, Panel B). In simulated vaginal fluid (Supplemental Table 5, Panel B1), Cr and Fe had the lowest average leaching fractions at 5% (95% CI not calculated) and 6% (95% CI: 3%–8%), respectively. Cu had the highest average at 233% (95% CI: −87% to 553%). In simulated menstrual fluid (Supplemental Table 5 Panel B2), Cr and Pb had the lowest average leaching fractions at 5% (95% CI: 5%–6%) and 5% (95% CI: 2%–7%), respectively. As and Ni had the highest leaching fractions at 48% (95% CI: 31%–65%) and 58% (95% CI: 38%–79%), respectively.

## Discussion

### Mechanisms of metal leaching and the role of pH and fluid composition

Our findings demonstrate that tampons can release metals under in vitro, physiologically relevant test conditions. Using leaching protocols that controlled for pH, temperature, and duration of tampon wear, we found that 14 metals tested leached from tampons. Metals such as As, Cd, and Pb exhibited larger release in simulated vaginal fluid (pH 4.2), while Ni and V showed greater leaching in simulated menstrual fluid (pH 7.4). Some metals, such as V, Hg, and As, had more leaching values below the MDL in menstrual fluid compared to vaginal fluid, suggesting minimal leaching under less acidic conditions. These differences demonstrate the central role of pH acidic environments weaken metal–material binding by increasing proton competition, thereby enhancing desorption and solubility.^35^^,^^36^ This mechanism is particularly relevant during light menstrual flow, when vaginal pH remains low, and tampons may be worn for extended durations, increasing the likelihood of mucosal exposure.

The chemical behavior of metals in biological fluids is largely determined by pH, ionic strength, and matrix interactions. In general, acidic environments weaken metal–material binding by increasing proton competition, thereby enhancing desorption and solubility of metals.^37^ Our findings align with well-documented chemical behavior: metals such as Cd, Cu, Pb, and Zn exhibit a “v-shaped” pH-dependent leaching curve, with elevated solubility at low and high pH and reduced mobility near neutral pH.^38^ However, other fluid properties, including ionic strength, protein content, and temperature, also modulate leaching. Furthermore, the presence of endogenous metals in menstrual fluid such as Fe, Mn, and Ca may reduce the chemical gradient^39^ that drives leaching from tampon materials. In other words, the presence of Fe in the simulated menstrual fluid may reduce net Fe release from the tampon, although the underlying mechanism was not evaluated in this study. This highlights how the composition of the surrounding medium, including its metal content, can modulate the mobility and bioaccessibility of metals in tampon materials.

Leaching behavior varied substantially by product characteristics, specifically organic composition and absorbency size. Nonorganic tampons had higher Cd, Co, Ni, Pb, and Zn concentrations and leached greater quantities of these metals in both simulated vaginal and menstrual fluids compared to organic tampons. Variation in ingredients and manufacturing processes could explain the different metal leaching profiles. While organic tampons are made with 100% organic cotton, nonorganic tampons may contain fragrances or synthetic fibers, and their cotton may be conventionally grown. Regarding tampon size, super-absorbency tampons leached greater amounts of all detectable metals compared to regular-absorbency tampons. Tampon sizes are classified by absorbency, the amount of fluid retained by the tampon. Yet, there is also a physical size difference between regular and super-absorbency tampons. The greater released mass from super-absorbency tampons may partly reflect differences in product mass, surface area, material composition, and the larger saturation volume used for these products. Understanding the sources of trace metals in tampons would help guide regulation to reduce metal exposure for tampon users.

Total metal content, as reported by Shearston et al.^5^ and the leached metal amounts measured in the present study provide complementary information about the presence and potential release of metals from tampon products. Low leachate percentages likely reflect strong matrix retention or limited solubility, while values exceeding 100%—as observed for As, Ba, and Cu vaginal fluid and Ni in menstrual fluid—can be explained by analytical uncertainty, unit-to-unit variability, or preferential desorption of loosely bound surface-associated metals. These findings confirm that total metal content alone is insufficient to assess exposure risk. Rather, accurate risk assessment must consider the interplay of fluid chemistry, material composition, and physiological conditions. Our study points to pH-dependent desorption and matrix interactions as key mechanisms of tampon-associated metal release and highlights the urgent need for standardized safety evaluations that reflect the complexity of real-world use.

### Comparison to previous studies

Our study addresses ongoing debates in the literature about metal exposure from menstrual products, particularly tampons. Shearston et al^5^ used total acid digestion to quantify the full metal content in tampons and highlighted potential health concerns based on high levels of Pb, As, and Cd. Marcelis et al^25^ conducted leaching tests with simulated fluids following ISO 10993 guidelines on tampons, menstrual cups, and menstrual sea sponges, concluding that metal exposure via tampons is negligible. However, their findings were reported in fluid-volume–normalized concentrations (ppb), which are influenced by the volume of leaching fluid used and do not directly indicate the total mass of metal released.

Our study integrates these perspectives by (1) using realistic fluid chemistry and tampon saturation protocols, and (2) assessing the percentage of leached metal from total metals. This enabled calculation of the leaching fraction—a crucial metric for evaluating potential exposure. Our results show that while total metal content identifies targets of concern, leaching data chatacterize the amount released under specific simulated-use conditions. The discrepancy between high total metal content and low leaching for some elements (eg, Fe) underscores the importance of including both metrics. This integrative approach provides a more complete understanding of exposure potential. Merely detecting the presence of a metal in a product is insufficient to assess the health risk, and measuring leached amounts alone provides limited insight without knowing what proportion of the total metal content they represent. Measuring total metal content establishes an absolute upper bound for potential exposure, particularly under atypical conditions such as improper storage (eg, prolonged heat exposure during transportation) that may increase leaching.

Our study extends Marcelis et al^25^ by providing quantitative, tampon-specific estimates of metal release under two simulated physiological conditions. Marcelis et al used a menstrual-fluid simulant with a final pH of 6.7 and sampled excess fluid. In contrast, we separately evaluated simulated vaginal fluid (pH 4.2) and menstrual fluid (pH 7.4), which contained a broader range of physiologically relevant constituents, and used product-specific saturation volumes. Their pilot study analyzed 11 metals in five samples from the combined tampon and sanitary-pad category. Tampons and pads were not tested in triplicate, and product-specific metal concentrations, variability, and statistical results were not reported, while our study evaluated 22 tampon products and 15 metals, corrected for fluid background concentrations, and reported mass released per tampon, leaching fractions (%), 95% CIs, and duplicate precision.

Our findings are broadly consistent with a recent US Food and Drug Administration (FDA) study,^40^ that measured metal release from tampons and conducted a toxicological risk assessment. Both studies detected several metals, including As, Cd, and Pb, in tampon extracts, which provides independent evidence that metals can be released from tampons under different experimental conditions. For most comparable metals, the median extracted concentrations in our study were higher than those reported by Jin et al.^40^ (Supplemental Table 3 and 7). However, these values should not be interpreted as direct estimates of relative leaching percentages because the data reported by Jin et al. did not permit calculation of the percentage of total metal content released. Direct comparison is also limited by differences in experimental conditions. Jin et al^40^ used acidified water as the extraction medium at 50°C, whereas we used simulated vaginal and menstrual fluids designed to better approximate physiological conditions. Differences in tampon products or in the ionic composition, viscosity, and protein components of simulated fluids could have resulted in different leaching profiles between our study, Marcelis et al^25^ and Jin et al.^40^ These differences emphasize the need for further testing, especially with larger sample sizes, to clarify the range of metal leaching from tampons.

### Simulated metal leaching from tampons and inferences for health risks

Cd and Pb are of particular concern among the leached metals due to their ubiquity and well-established toxicity. Cd can cause reproductive, hepatic, hematological, and immunological effects,^41^ and functions as a metalloestrogen by binding estrogen receptors, potentially contributing to hormone-dependent cancers such as breast and uterine cancer.^42^ No Pb exposure level is considered safe; Pb has the highest disease burden in disability-adjusted life years, exceeding PFAS, phthalates, and PCBs.^43^ Both Pb and Cd are recognized by the American Heart Association as cardiovascular risk factors,^44^ with Pb alone linked to ∼250,000 cardiac deaths annually.^45^ Notably, cellular Cd and Pb uptake is enhanced under Fe-depleted conditions, as both metals compete with Fe for transport via divalent metal transporter-1 (e.g., DMT-1).^46^ Given that Fe levels decline across the menstrual cycle with depletion most pronounced during and immediately after menstruation,^47^ Cd and Pb bioaccessibility at the vaginal epithelium may vary by cycle phase.

It is important to emphasize that the metals quantified here represent amounts leached into simulated fluid under in vitro conditions and should not be equated with confirmed human exposure: whether and to what extent these metals are absorbed across the vaginal epithelium is the critical next research step. While direct evidence of systemic metal absorption from tampons remains to be established, the literature on copper-containing intrauterine devices (IUDs) offers a relevant biological parallel. Although tampons and Cu-IUDs differ in use patterns, tampons are used intermittently in the vagina during menstruation and Cu-IUDs reside in the uterine cavity and continuously release Cu for years as part of their contraceptive function, both involve potential metal exposure at reproductive mucosa. A few studies reported increases in blood or serum Cu concentrations among Cu-IUD users,^48^^,^^49^ though others were inconclusive. Nevertheless, these findings still suggest that metals released from devices in intimate mucosal contact can reach systemic circulation and shows the need to study whether tampon-associated metal leaching poses a similar pathway.

To further contextualize the leached quantities within established regulatory frameworks, we compared them with additional health-based benchmarks. The US Environmental Protection Agency (EPA) sets maximum contaminant levels (MCLs) in drinking water at 10 parts per billion (ppb) for As and 5 ppb for both Cd and an action level of 15 ppb for Pb with an MCL goal of zero.^50^ Based on average leaching values (ng) into simulated fluids, lifetime tampon use—estimated at 10,000 products over 40 reproductive years—could result in cumulative exposures of approximately 63,100 to 91,500 ng of As, 46,400 to 49,900 ng of Cd, and 156,000 to 403,000 ng of Pb. As a reference estimate and not accounting for first-pass liver metabolism, these amounts are equivalent to ingesting ∼6 to 9 liters of drinking water at the EPA’s MCL for As, ∼10 liters for Cd, and ∼10 to 27 liters for Pb at the current Pb action level. While these comparisons are not direct risk assessments, they provide an important reference point: if vaginal absorption occurs, repeated low-level exposure could contribute to cumulative metal body burden; however, absorption was not evaluated in this study. Given the frequency, duration, and intimate nature of tampon use, these findings highlight the urgent need for regulatory scrutiny, systematic safety testing, and deeper investigation into the health effects of chronic low-dose vaginal exposure to toxic metals. Converting the estimated cumulative lifetime leached amounts to a daily equivalent (assuming 5 tampon-days per cycle, 12 cycles per year, over 40 reproductive years, yielding ∼2400 days of tampon use), the average daily leached As, Cd, and Pb from tampon use are approximately 26 to 38 ng/day, 19 to 21 ng/day, and 65 to 168 ng/day, respectively. However, there is no identified threshold below which adverse neurological effects do not occur. The detection of Pb in tampon leachates warrants attention because WHO states that no safe blood lead concentration has been identified^51^, which supports efforts to minimize Pb exposure from consumer products. This is particularly relevant because Pb is among the metals detected in the leacheates of both simualted vaginal and menstrual fluid. Even under the FDA study’s assumption of 100% vaginal absorption, Pb had second-lowest margins of safety among the detected metals after antimony.^40^ Although this value exceeded the study’s threshold of 1 and was interpreted as indicating low toxicological concern, it placed Pb among the metals closest to the tolerable-intake benchmark. A recent study integrated experimental data on Pb partitioning in menstrual fluid with mechanistic modeling and estimated that under a 4-hour wear scenario, less than 1 ng and less than 0.3% of theoretically released Pb would be available for vaginal tissue uptake.^52^ While this suggests the fraction of leached Pb reaching vaginal tissue may be very small, the study used only a single metal and a single wear duration, and did not address the cumulative effect of repeated exposures over a reproductive lifetime.

### Limitations and future research

While our study used physiologically relevant conditions and included a broad array of tampon products, several limitations should be mentioned. We analyzed 22 products spanning multiple brands and absorbencies, but the sample size limited statistical power and may have obscured subtle differences in metal leaching. Not all tampon types were tested across both simulated fluids, as we prioritized consistency in lot and batch numbers. Future studies should investigate metal content and leaching from other types of menstrual products, given the high usage of disposable menstrual pads among women^53^^,^^54^ and findings of PFAS in menstrual cups.^9^

Apparent recoveries exceeding 100% likely reflect analytical uncertainty, sample variability, and differences in tampon surface binding. Measurements of total vs leached metals were performed on separate tampon units, consistent with Shearston et al,^5^ leading to minor discrepancies. Surface characteristics may also affect metal binding and release, with loosely bound surface metals more readily leached. Minor handling inconsistencies or exposure differences could also contribute.

Although our study simulated realistic leaching conditions, we did not assess whether metals are absorbed through the vaginal mucosa. Absorption may vary by metal species, chemical form, and local tissue physiology. Importantly, chemicals entering via the vaginal epithelium reach the vaginal, uterine, and ovarian blood supply directly, bypassing hepatic first-pass metabolism^55^^,^^56^ Animal studies also support substantial vaginal uptake; for example, silver nanoparticles administered intravaginally in rabbits accumulated in reproductive tissues and entered systemic circulation.^57^ These mechanisms raise concern that tampon-derived metals could accumulate in reproductive organs, where toxic metals have been linked to gynecological disorders.^58^ One study, for instance, reported 4-fold higher mercury in hyperplastic compared to normal endometrial tissue.^59^ Future studies should evaluate the bioaccessibility of leached metals using in vivo or in vitro models to clarify potential health risks and inform safety standards for menstrual products.

Lastly, our simulated leaching method did not fully reproduce the dynamic vaginal environment, including variable fluid flow, protein and cellular binding, product reabsorption, potential tissue uptake, and vaginal microbiota. Because of the complexity of in vivo conditions and because we cannot generalize the behavior of all leached metals, it is not clear whether our results represent an over- or under-estimation of metal leaching in vivo. Comparison of in vitro and in vivo metal leaching in orthodontic appliances found that certain metal ions leached more under in vitro conditions (eg, Fe) while others leached more in vivo (eg, Cr and Ti).^60^ Similarly, improved in vitro models that replicate unique aspects of the in vivo environment (eg, microbiota, fluid clearance) show that microbiota may increase the bioaccessibility of some metals (eg, Zn, Ni, Cd) and decrease that of others (eg, Cu).^61^ The use of fixed leaching volumes in static in vitro models may underestimate metal leaching if the accumulation of metal ions in the leachate reduces the concentration gradient that drives the leaching.^62^^,^^63^ The results of this study should be interpreted as metal release under the specified experimental conditions rather than as predicted in vivo exposure. We emphasize the need for future in vivo studies of metal leaching in the vaginal environment.

## Conclusions

We assessed metal leaching from tampons using simulated vaginal and menstrual fluids and in vitro, physiologically relevant conditions to evaluate the potential for bioavailability. We found that tampons released measurable amounts of multiple metals into simulated vaginal and menstrual fluids under the specified in vitro conditions. Release differed across fluid composition and product characteristics, and total metal content did not predict the pote amount released. These findings confirm that metal release from tampons can occur but do not determine the fraction absorbed through vaginal tissue, the resulting internal dose, or health risk. Studies incorporating vaginal tissue models, fluid turnover, metal speciation, and toxicokinetics are needed to determine whether tampon-associated metal release contributes meaningfully to human exposure.

## Condensation

Tampons are used by approximately 100 million people worldwide to manage their menstruation. Although metals have been detected in tampons, it is unclear whether they leach under physiological conditions and whether changes in vaginal pH during menstruation influence leachability. Here we show that tampons leach measurable amounts of 14 metals, including lead, arsenic, and cadmium, into simulated vaginal and simulated menstrual fluid under physiologically relevant conditions. We found that leaching was generally higher in vaginal fluid compared to menstrual fluid. Metal leaching varied by brand, tampon composition, and absorbency capacity. We then calculated the leaching fraction—the proportion of total metal content in the tampon leached. This is a useful metric for understanding potential exposure. By quantifying metal leaching from tampons using realistic fluid chemistry and tampon saturation protocols, our findings highlight the need to investigate vaginal absorption of metals from tampons and potential health effects.

## CRediT authorship contribution statement

**Gillian S. Kaneshiro:** Writing – original draft, Visualization, Formal analysis. **Daysi Martinez:** Writing – review & editing, Visualization, Formal analysis. **Heidi Hannoush:** Writing – review & editing, Visualization, Data curation. **Marianthi Anna Kioumourtzoglou:** Writing – review & editing, Supervision, Funding acquisition. **Jenni A. Shearston:** Writing – review & editing, Investigation. **Nancy Reame:** Writing – review & editing, Supervision. **Kristen Upson:** Writing – review & editing, Supervision, Funding acquisition. **Kathrin Schilling:** Writing – original draft, Supervision, Methodology, Funding acquisition, Conceptualization.

## Appendix Supplementary materials

Image, application 1

## Footnotes

Supplementary material associated with this article can be found in the online version at doi:10.1016/j.xagr.2026.100686.
