# Supplementary material for: In vitro metal leaching from tampons under simulated vaginal and menstrual pH conditions
Source: AJOG Glob Rep. 2026 Aug 25;6(4):100686. doi: 10.1016/j.xagr.2026.100686 (PMC13632032; doi:10.1016/j.xagr.2026.100686)
Supplement: Supplementary file 1 [file mmc1.docx]

**Supplemental Information**

***for***

**In vitro Metal Leaching from Tampons Under Simulated Vaginal and Menstrual pH Conditions**

**Authors:** Gillian S. Kaneshiro^1*^, Daysi Martinez^1*^, Heidi Hannoush^1^, Marianthi Anna Kioumourtzoglou^1,2,3^, Jenni A. Shearston^4^, Nancy Reame^5^, Kristen Upson^6^, Kathrin Schilling^1^

**Author Details:**

^1^Department Environmental Health Sciences, Mailman School of Public Health, Columbia University, New York, NY 10032, USA;

^2^Department of Epidemiology, School of Public Health, Brown University, Providence, RI 02903, USA;

^3^Institute at Brown for the Environment and Society, Brown University, Providence, RI 02903, USA;

^4^Department of Integrative Physiology, University of Colorado Boulder, CO 80309, USA;

^5^Columbia University School of Nursing, New York, NY 10032, USA**;**

^6^Department of Epidemiology and Biostatistics, College of Human Medicine, Michigan State University, East Lansing, MI 48824, USA

**Supplemental Table 1.** Background metal concentrations in simulated vaginal fluid (BZ181) and simulated menstrual fluid (BZ266) obtained from Biochemazone. Metal concentrations of instrument blanks are also reported.

***Panel A. Simulated vaginal fluid (BZ181)***

| **Metal** | **Regular absorbency** | | | **Super absorbency** | | |
| --- | --- | --- | --- | --- | --- | --- |
|  | **n** | **Mean (ng/mL)** | **RSD (%)** | **n** | **Mean (ng/mL)** | **RSD (%)** |
| **As†** | 4 | 0.464 | 19 | 3 | 0.472 | 23 |
| **Ba** | 4 | 7.58 | 2 | 4 | 7.29 | 7 |
| **Cd†** | 4 | 0.040 | 28 | 4 | 0.033 | 47 |
| **Co** | 4 | 0.061 | 14 | 4 | 0.057 | 24 |
| **Cr** | 4 | 0.504 | 4 | 4 | 0.417 | 10 |
| **Cu†** | 3 | 0.956 | 24 | 4 | 0.698 | 17 |
| **Fe** | 4 | 6.30 | 5 | 4 | 5.66 | 8 |
| **Hg†** | 4 | −0.003 | NC | 4 | 0.111 | 173 |
| **Mn** | 3 | 0.179 | 12 | 4 | 0.229 | 14 |
| **Ni** | 4 | 3.93 | 5 | 4 | 3.67 | 9 |
| **Pb** | 4 | 0.250 | 8 | 4 | 0.249 | 12 |
| **Se†** | 4 | 0.246 | 135 | 4 | 0.485 | 75 |
| **Sr** | 4 | 50.4 | 1 | 4 | 49.1 | 5 |
| **V** | 4 | 0.114 | 10 | 4 | 0.104 | 6 |
| **Zn** | 3 | 5.18 | 9 | 4 | 3.49 | 9 |

***Panel B. Simulated menstrual fluid (BZ266)***

| **Metal** | **Regular absorbency** | | | **Super absorbency** | | |
| --- | --- | --- | --- | --- | --- | --- |
|  | **n** | **Mean (ng/mL)** | **RSD (%)** | **n** | **Mean (ng/mL)** | **RSD (%)** |
| **As†** | 3 | 0.031 | 42 | 3 | 0.033 | 56 |
| **Ba** | 4 | 32.4 | 1 | 3 | 32.6 | 1 |
| **Cd†** | 4 | 0.015 | 28 | 3 | 0.013 | 188 |
| **Co** | 4 | 0.035 | 29 | 3 | 0.043 | 5 |
| **Cr** | 4 | 1.64 | 2 | 3 | 1.61 | 2 |
| **Cu†** | 4 | 13.5 | 32 | 3 | 13.2 | 39 |
| **Fe** | 4 | 425 | 4 | 3 | 419 | 5 |
| **Hg†** | 4 | 0.064 | 42 | 3 | 0.042 | 70 |
| **Mn** | 4 | 2.20 | 3 | 3 | 2.18 | 0.5 |
| **Ni** | 4 | 19.9 | 2 | 3 | 20.2 | 0.3 |
| **Pb** | 4 | 0.042 | 21 | 3 | 0.041 | 6 |
| **Se†** | 4 | 0.305 | 34 | 3 | 0.287 | 50 |
| **Sr** | 4 | 12.7 | 3 | 3 | 12.7 | 5 |
| **V** | 4 | 0.721 | 3 | 3 | 0.728 | 2 |
| **Zn** | 4 | 34.9 | 8 | 3 | 34.0 | 3 |

**Panel A and B Note.** Values are background metal concentrations measured in simulated fluid blanks associated with the regular- and super-absorbency experimental groups. The cell-specific n values indicate the number of measurements used to calculate each mean and relative standard deviation. †One or more individual measurements were below the MDL in one or both simulated fluids. The RSD for Hg in the vaginal-fluid regular-absorbency group was not calculated because the blank-corrected mean was negative. MDL = method detection limit; NC = not calculated; RSD = relative standard deviation.

***Panel C. Instrument acid blanks***

| **Metal** | **n** | **Mean (ng/mL)** | **SD (ng/mL)** | **LOD** | **MDL regular (ng)** | **MDL super (ng)** |
| --- | --- | --- | --- | --- | --- | --- |
| **As** | 14 | 0.009 | 0.047 | 0.155 | 1.63 | 2.48 |
| **Ba** | 14 | 0.010 | 0.017 | 0.055 | 0.581 | 0.886 |
| **Cd** | 14 | -0.001 | 0.007 | 0.022 | 0.229 | 0.349 |
| **Co** | 14 | -0.007 | 0.003 | 0.010 | 0.102 | 0.156 |
| **Cr** | 14 | 0.000 | 0.030 | 0.101 | 1.06 | 1.61 |
| **Cu** | 12 | 1.14 | 1.65 | 5.48 | 57.6 | 87.7 |
| **Fe** | 14 | 0.271 | 0.593 | 1.98 | 20.8 | 31.6 |
| **Hg** | 13 | 0.044 | 0.054 | 0.181 | 1.90 | 2.89 |
| **Mn** | 14 | -0.008 | 0.015 | 0.052 | 0.541 | 0.825 |
| **Ni** | 14 | -0.046 | 0.048 | 0.158 | 1.66 | 2.54 |
| **Pb** | 14 | -0.004 | 0.006 | 0.021 | 0.225 | 0.343 |
| **Se** | 14 | -0.119 | 0.203 | 0.676 | 7.10 | 10.82 |
| **Sr** | 14 | 0.020 | 0.038 | 0.126 | 1.33 | 2.02 |
| **V** | 14 | 0.008 | 0.005 | 0.018 | 0.192 | 0.293 |
| **Zn** | 14 | 0.152 | 0.241 | 0.802 | 8.42 | 12.84 |

**Panel C Note.** Values are metal concentrations measured in instrument acid blanks. The LoD was defined as 3.33 times the standard deviation of blank measurements. The MDL are the LoD scaled by the saturation volume (10.5 mL for regular absorbency, 16.0 mL for super absorbency). MDL = method detection limit; SD = standard deviation; LoD = limit of detection.

**Supplemental Table 2.** Summary statistics for the amounts of 15 metals leached from tampons into simulated vaginal fluid (nanograms).

|  | **As** | **Ba** | **Cd** | **Co** | **Cr** | **Cu** | **Fe** | **Hg** | **Mn** | **Ni** | **Pb** | **Se** | **Sr** | **V** | **Zn** |
| --- | --- | --- | --- | --- | --- | --- | --- | --- | --- | --- | --- | --- | --- | --- | --- |
| **N above MDL** | 10 | 18 | 18 | 18 | 18 | 3 | 18 | 4 | 18 | 18 | 16 | 0 | 16 | 13 | 18 |
| **Minimum** | 2.05 | 54.4 | 0.868 | 1.25 | 3.81 | 118.0 | 40.2 | 3.51 | 16.3 | 8.06 | 0.455 | NC | 31.6 | 0.349 | 351 |
| **25^th^ Percentile** | 4.01 | 500 | 1.83 | 2.72 | 7.20 | 142.42 | 135 | 3.798 | 64.0 | 16.4 | 4.74 | NC | 87.2 | 0.465 | 4496 |
| **50^th^ Percentile** | 7.26 | 979 | 3.33 | 8.29 | 10.5 | 166.9 | 382 | 9.652 | 276 | 40.1 | 9.76 | NC | 195 | 0.907 | 12457 |
| **75^th^ Percentile** | 11.9 | 2097 | 5.89 | 14.9 | 17.0 | 508.9 | 487 | 18.37 | 442 | 105 | 61.6 | NC | 523 | 1.59 | 30024 |
| **Maximum** | 23.4 | 4427 | 21.6 | 23.2 | 35.9 | 851 | 2384 | 27.3 | 1792 | 341 | 267 | NC | 2981 | 24.9 | 107124 |
| **Mean** | 9.15 | 1430 | 4.99 | 10.1 | 13.6 | 378.6 | 460 | 12.52 | 338 | 81.9 | 40.3 | NC | 556 | 4.50 | 22863 |
| **SD** | 6.97 | 1298 | 5.38 | 7.56 | 8.78 | 410 | 530 | 11.27 | 409 | 95.9 | 68.2 | NC | 885 | 8.80 | 27574 |
| **95% CI** | (4.84, 13.5) | (830, 2029) | (2.51, 7.48) | (6.57, 13.6) | (9.58, 17.7) | (-85.1, 842) | (215, 705) | (1.47, 23.6) | (149, 527) | (37.6, 126) | (6.94, 73.7) | NC | (122, 989) | (-0.283, 9.28) | (10124, 35601) |
| **MDL**  **Regular (ng)** | 1.63 | 0.581 | 0.229 | 0.102 | 1.06 | 57.6 | 20.8 | 1.90 | 0.54 | 1.66 | 0.22 | 7.10 | 1.33 | 0.19 | 8.42 |
| **MDL**  **Super (ng)** | 2.48 | 0.886 | 0.349 | 0.156 | 1.61 | 87.7 | 31.6 | 2.89 | 0.83 | 2.54 | 0.34 | 10.82 | 2.02 | 0.29 | 12.84 |
| **Samples below MDL** | 8 | 0 | 0 | 0 | 0 | 15 | 0 | 14 | 0 | 0 | 2 | 18 | 2 | 5 | 0 |
| **% of samples below MDL** | 44% | 0% | 0% | 0% | 0% | 83% | 0% | 78% | 0% | 0% | 11% | 100% | 11% | 28% | 0% |

**Note.** Values below the method detection limit (MDL) were excluded from the summary statistics. The value of n represents the number of samples at or above the MDL that were included in the calculations. NC indicates that a statistic was not calculated because no measurements were at or above the MDL. All 18 measurements of Se were below the MDL. MDLs differed between regular- and super-absorbency tampons. CI = confidence interval; NC = not calculated; SD = standard deviation.

**Supplemental Table 3.** Summary statistics for the amounts of 15 metals leached from tampons into simulated menstrual fluid (nanograms).

|  | **As** | **Ba** | **Cd** | **Co** | **Cr** | **Cu** | **Fe** | **Hg** | **Mn** | **Ni** | **Pb** | **Se** | **Sr** | **V** | **Zn** |
| --- | --- | --- | --- | --- | --- | --- | --- | --- | --- | --- | --- | --- | --- | --- | --- |
| **N above MDL** | 6 | 18 | 16 | 18 | 18 | 3 | 0 | 0 | 18 | 18 | 14 | 0 | 18 | 4 | 18 |
| **Minimum** | 2.16 | 37.0 | 0.524 | 0.304 | 4.05 | 74.2 | NC | NC | 13.8 | 23.4 | 0.534 | NC | 16.8 | 0.795 | 592 |
| **25^th^ Percentile** | 3.09 | 160 | 1.22 | 2.46 | 7.37 | 89.04 | NC | NC | 249 | 83.9 | 1.993 | NC | 27.7 | 0.988 | 1682 |
| **50^th^ Percentile** | 4.75 | 272 | 3.07 | 6.72 | 10.2 | 103.9 | NC | NC | 320 | 118 | 10.42 | NC | 57.0 | 6.38 | 7346 |
| **75^th^ Percentile** | 9.31 | 544 | 6.72 | 9.92 | 13.0 | 106.5 | NC | NC | 466 | 162 | 21.8 | NC | 385 | 12.91 | 17221 |
| **Maximum** | 12.8 | 1090 | 15.1 | 19.7 | 18.5 | 109 | NC | NC | 907 | 282 | 50.6 | NC | 3057 | 16.5 | 39492 |
| **Mean** | 6.31 | 364 | 4.64 | 7.18 | 10.3 | 95.7 | NC | NC | 341 | 123 | 15.6 | NC | 371 | 7.51 | 11800 |
| **SD** | 4.39 | 272 | 4.44 | 5.77 | 4.40 | 18.8 | NC | NC | 228 | 68.9 | 16.6 | NC | 725 | 7.86 | 12227 |
| **95% CI** | (2.80, 9.82) | (238, 490) | (2.47, 6.82) | (4.51, 9.84) | (8.29, 12.4) | (74.4, 117) | NC | NC | (236, 446) | (91.6, 155) | (6.91, 24.3) | NC | (35.7, 706) | (-0.185, 15.2) | (6151, 17449) |
| **MDL**  **Regular (ng)** | 1.63 | 0.581 | 0.229 | 0.102 | 1.06 | 57.6 | 20.8 | 1.9 | 0.54 | 1.66 | 0.22 | 7.10 | 1.33 | 0.19 | 8.42 |
| **MDL**  **Super (ng)** | 2.48 | 0.886 | 0.349 | 0.156 | 1.61 | 87.7 | 31.6 | 2.89 | 0.83 | 2.54 | 0.34 | 10.82 | 2.02 | 0.29 | 12.84 |
| **Samples below MDL** | 12 | 0 | 2 | 0 | 0 | 15 | 18 | 18 | 0 | 0 | 4 | 18 | 0 | 14 | 0 |
| **% of samples below MDL** | 67% | 0% | 11% | 0% | 0% | 83% | 100% | 100% | 0% | 0% | 22% | 100% | 0% | 78% | 0% |

**Note.** Values below the method detection limit (MDL) were excluded from the summary statistics. The value of n represents the number of samples at or above the MDL that were included in the calculations. NC indicates that a statistic was not calculated because no measurements were at or above the MDL. All 18 measurements of Fe, Hg, and Se were below the MDL. MDLs differed between regular- and super-absorbency tampons. CI = confidence interval; NC = not calculated; SD = standard deviation.

**Supplemental Table 4.** Analytical precision of metal measurements in tampon leachates, expressed as the mean relative standard deviation (RSD) of replicate measurements. The number of samples below the method detection limit (MDL) and the number of samples contributing to each mean RSD are also reported.

| **Metal** | **Vaginal fluid (n=4)** | | | **Menstrual fluid (n=8)** | | |
| --- | --- | --- | --- | --- | --- | --- |
|  | **Mean RSD** | **n with RSD** | **n < MDL** | **Mean RSD** | **n with RSD** | **n < MDL** |
| **As** | 62% | 2 | 2 | - | 0 | 7 |
| **Ba** | 18% | 4 | 0 | 19% | 7 | 1 |
| **Cd** | 33% | 4 | 0 | 56% | 6 | 1 |
| **Co** | 44% | 4 | 0 | 27% | 7 | 0 |
| **Cr** | 10% | 4 | 0 | 16% | 7 | 1 |
| **Cu** | - | 0 | 3 | - | 0 | 7 |
| **Fe** | 40% | 4 | 0 | - | 0 | 8 |
| **Hg** | - | 0 | 3 | - | 0 | 8 |
| **Mn** | 31% | 4 | 0 | 17% | 7 | 0 |
| **Ni** | 46% | 4 | 0 | 21% | 7 | 1 |
| **Pb** | 39% | 4 | 0 | 25% | 6 | 1 |
| **Se** | - | 0 | 4 | - | 0 | 8 |
| **Sr** | 23% | 4 | 0 | 25% | 7 | 0 |
| **V** | 41% | 4 | 0 | 33% | 1 | 7 |
| **Zn** | 61% | 4 | 0 | 42% | 7 | 0 |

**Note.** RSD = relative standard deviation; MDL = method detection limit (see Table S2 for MDLs). Four tampons in vaginal fluid and eight in menstrual fluid were tested in duplicate. A sample was classified as below the MDL when the average of its two replicates fell below the metal- and absorbency-specific MDL. Samples in which one replicate was above and the other below the MDL were not assigned an RSD and were excluded from the mean; "n with RSD" reports how many samples contributed to each mean. A dash indicates that no sample was eligible.

**Supplemental Table 5.** Total metal concentrations and leaching fractions for matched brand-product line-absorbency combinations.

**Panel A.** Geometric mean total metal concentrations (ng/g) for 16 unique brand-product line-absorbency combinations among 20 tampons that were used to calculate leaching fractions. Reproduced from Shearston et al. (2024).

| Brand | Product Line | Number of Tampons | Number of Samples | Absorbency | As | Ba | Cd | Co | Cr^a^ | Cu | Fe | Hg^a^ | Mn | Ni | Pb | Se | Sr | V | Zn |
| --- | --- | --- | --- | --- | --- | --- | --- | --- | --- | --- | --- | --- | --- | --- | --- | --- | --- | --- | --- |
| A | 1 | 1 | 2 | Regular | 2.99 | 1,244 | 14.5 | 24.0 | - | 37.4 | 4,164 | 4.9 | 434 | 36.9 | 170 | 18.3 | 243 | 6.06 | 78,411 |
| A | 3 | 2 | 4 | Regular | 2.39 | 1,167 | 13.8 | 35.9 | - | 43.0 | 2,484 | - | 336 | 55.7 | 168 | 5.76 | 175 | 4.1 | 70,973 |
| A | 3 | 2 | 4 | Super | 2.6 | 1,473 | 15.7 | 27.4 | - | 58.3 | 3,243 | 2.3 | 376 | 61.0 | 204 | 7.49 | 236 | 5.19 | 83,522 |
| B | 4 | 1 | 3 | Super | 1.53 | 3,942 | 9.43 | 22.8 | - | 206 | 1,721 | - | 165 | 102 | 211 | 241 | 143 | 4.77 | 48,271 |
| C | 5 | 2 | 3 | Regular | 1.61 | 159 | 5.55 | 15.9 | - | 89.8 | 3,586 | - | 350 | 70.7 | 103 | 11.1 | 205 | 5.16 | 65,783 |
| C | 5 | 2 | 4 | Super | 1.44 | 1,524 | 8.63 | 25.9 | - | 55.3 | 1,480 | - | 363 | 88.2 | 243 | 9.71 | 76.5 | 2.5 | 68,712 |
| D | 7 | 1 | 2 | Super | 12.1 | 580 | 1.98 | 7.08 | - | 73.6 | 14,986 | - | 1,000 | 67.0 | 49.5 | 9.6 | 1,460 | 34.1 | 24,057 |
| E | 8 | 1 | 2 | Regular | 5.05 | 2,258 | 11.4 | 17.8 | - | 44.4 | 1,645 | - | 880 | 66.4 | 171 | 9.7 | 95.5 | 3.15 | 155,992 |
| E | 8 | 1 | 2 | Super | 3.89 | 1,986 | 11.3 | 24.1 | - | 39.2 | 1,045 | - | 331 | 80.5 | 272 | 10.4 | 87.4 | 2.81 | 70,269 |
| F | 9 | 1 | 2 | Super | 6.9 | 201 | 1.32 | 6.44 | 85.3 | 51.5 | 16,658 | - | 424 | 99.9 | 48.9 | 29.1 | 461 | 29.8 | 18,255 |
| G | 10 | 1 | 2 | Super | 6.36 | 341 | 2.57 | 12.9 | 95.5 | 76.7 | 23,820 | - | 669 | 106 | 89.6 | 25.8 | 428 | 56.9 | 24,251 |
| J | 14 | 1 | 2 | Super | 1.04 | 7,946 | 4.66 | 14.9 | - | 151 | 1,565 | - | 39.1 | 124 | 48.6 | 758 | 250 | 4.29 | 37,355 |
| K | 15 | 1 | 2 | Super | 1.56 | 7,513 | 4.79 | 25.3 | - | 171 | 1,707 | - | 53.9 | 157 | 50.5 | 1,120 | 223 | 4.4 | 40,441 |
| L | 16 | 1 | 2 | Regular | 2.15 | 5,617 | 1.33 | 11.3 | - | 821 | 1,315 | - | 64.5 | 136 | 60.6 | 671 | 186 | 8.16 | 23,595 |
| M | 17 | 1 | 2 | Regular | 5.38 | 210 | 2.86 | 6.83 | - | 45.5 | 14,756 | - | 532 | 70.0 | 43.5 | 13.1 | 371 | 26.1 | 14,917 |
| N | 18 | 1 | 2 | Super | 1.8 | 360 | 2.11 | 5.68 | - | 141 | 9,170 | 2.7 | 279 | 47.9 | 33.0 | 6.71 | 1,362 | 14.1 | 30,662 |

**Panel A note.** Instrument-reported values were used for concentrations below the method detection limit (MDL). Overall, of the 60 tampon samples analyzed in Shearston et al. (2024), 54 of 60 Cr measurements (90.0%) and 55 of 60 Hg measurements (91.7%) were below the MDL. A dash indicates that the geometric mean was below the MDL.

**Panel B.** Leaching fractions of total metal content for tampons in simulated fluids.

*Panel B1. Simulated vaginal fluid*

| **Brand** | **Product Line** | **Absorbency** | **As** | **Ba** | **Cd** | **Co** | **Cr** | **Cu** | **Fe** | **Mn** | **Ni** | **Pb** | **Sr** | **V** | **Zn** |
| --- | --- | --- | --- | --- | --- | --- | --- | --- | --- | --- | --- | --- | --- | --- | --- |
| A | 1 | Regular | * | 28% | 52% | 33% | * | * | 1% | 46% | 42% | 28% | 54% | 5% | 43% |
| A | 3 | Regular | * | 29% | 16% | 15% | * | * | 1% | 38% | 12% | 17% | 63% | * | 21% |
| C | 5 | Regular | * | 307% | 39% | 75% | * | 74% | 6% | 49% | 46% | 45% | 9% | 5% | 18% |
| C | 5 | Super | 91% | 26% | 91% | 33% | * | 560% | 22% | 45% | 46% | 40% | 39% | 13% | 57% |
| D | 7 | Super | 57% | 36% | 39% | 28% | * | * | 5% | 53% | 10% | 3% | 50% | 21% | 11% |
| E | 8 | Super | * | 27% | 14% | 13% | * | * | 14% | 21% | 15% | 12% | 44% | 8% | 24% |
| F | 9 | Regular | 9% | 8% | 19% | 4% | * | * | 1% | 16% | 1% | 3% | 7% | 1% | 3% |
| F | 9 | Super | 64% | 33% | 61% | 14% | 5% | * | 1% | 42% | 6% | 5% | 59% | 3% | 2% |
| G | 10 | Regular | * | 36% | 33% | 26% | * | 65% | 1% | 42% | 15% | 10% | 39% | 24% | 18% |
| I | 12 | Regular | * | 17% | 5% | 14% | * | * | 7% | 19% | 18% | 3% | 22% | 4% | 20% |
| I | 12 | Super | 98% | 12% | 17% | 19% | * | * | 10% | 27% | 19% | 3% | 31% | 2% | 23% |
| J | 14 | Regular | * | 11% | 14% | 19% | * | * | 6% | 23% | 22% | 0.5% | * | * | 17% |
| J | 14 | Super | 414% | 16% | 57% | 33% | * | * | 9% | 38% | 83% | 1% | 25% | 3% | 20% |
| K | 15 | Regular | * | 13% | 16% | 13% | * | * | 3% | 13% | 14% | * | * | * | 12% |
| K | 15 | Super | 114% | 20% | 59% | 26% | * | * | 9% | 30% | 74% | 0.4% | 32% | * | 13% |
| L | 16 | Regular | 53% | 25% | 45% | 19% | * | * | 3% | 21% | 37% | * | 31% | * | 8% |
| M | 17 | Regular | 55% | 33% | 50% | 13% | * | * | 2% | 48% | 9% | 13% | 61% | 3% | 6% |
| N | 18 | Super | 323% | 41% | 37% | 7% | * | * | 2% | 53% | 6% | 8% | 72% | 3% | 2% |
| **Mean** | | | 128% | 40% | 37% | 22% | 5% | 233% | 6% | 35% | 26% | 12% | 40% | 7% | 18% |
| **95% CI Lower Limit** | | | 46% | 9% | 27% | 15% | NC | -87% | 3% | 28% | 15% | 5% | 30% | 3% | 11% |
| **95% CI Upper Limit** | | | 210% | 71% | 47% | 30% | NC | 553% | 8% | 41% | 37% | 19% | 49% | 11% | 24% |

Panel B1 note. Leaching fractions calculated for tampons saturated in simulated vaginal fluid (n=18). Tampons with total metals or with leachates below the method detection limit (MDL) are indicated with *. Hg and Se are not shown since all samples were below the MDL for either metal leachate or total metal data. NC indicates that a statistic was not calculated because of insufficient measurements at or above the MDL. CI = confidence interval; NC = not calculated.

*Panel B2. Simulated menstrual fluid*

| **Brand** | **Product Line** | **Absorbency** | **As** | **Ba** | **Cd** | **Co** | **Cr** | **Cu** | **Mn** | **Ni** | **Pb** | **Sr** | **V** | **Zn** |
| --- | --- | --- | --- | --- | --- | --- | --- | --- | --- | --- | --- | --- | --- | --- |
| A | 1 | Regular | * | 11% | 24% | 17% | * | * | 44% | 157% | 4% | 57% | * | 9% |
| A | 3 | Regular | * | 8% | 16% | 19% | * | * | 44% | 92% | 5% | 35% | * | 12% |
| A | 3 | Super | 45% | 15% | 27% | 18% | * | * | 52% | 107% | 4% | 51% | * | 11% |
| B | 4 | Super | * | 5% | 27% | 13% | * | * | 53% | 48% | 0.3% | 13% | * | 4% |
| C | 5 | Regular | * | 42% | 29% | 27% | * | * | 39% | 85% | 15% | 5% | * | 14% |
| C | 5 | Super | 76% | 8% | 44% | 24% | * | * | 64% | 90% | 7% | 24% | * | 21% |
| E | 8 | Regular | 27% | 4% | 27% | 20% | * | * | 32% | 111% | 7% | 11% | * | 11% |
| E | 8 | Super | * | 5% | 56% | 35% | * | * | 56% | 83% | 7% | 12% | * | 19% |
| F | 9 | Regular | 22% | 5% | 19% | 2% | * | * | 23% | 4% | 3% | 33% | 1% | 8% |
| F | 9 | Super | 64% | 13% | 22% | 10% | 5% | * | 48% | 16% | 5% | 75% | 2% | 4% |
| G | 10 | Regular | * | 13% | 13% | 5% | * | * | 20% | 12% | 2% | 21% | 13% | 1% |
| G | 10 | Super | 56% | 23% | 18% | 5% | 5% | 37% | 38% | 21% | 3% | 61% | 8% | 8% |
| I | 12 | Regular | * | 3% | * | 9% | * | 22% | 24% | 33% | * | 6% | * | 5% |
| I | 12 | Super | * | 3% | * | 8% | * | * | 29% | 30% | * | 8% | * | 5% |
| J | 14 | Regular | * | 3% | 8% | 12% | * | * | 24% | 28% | * | 7% | * | 3% |
| J | 14 | Super | * | 5% | 26% | 9% | * | * | 28% | 87% | 0.4% | 10% | * | 3% |
| K | 15 | Regular | * | 3% | 9% | 6% | * | 22% | 11% | 21% | * | 3% | * | 4% |
| N | 18 | Super | * | 38% | 20% | 2% | * | * | 50% | 25% | 1% | 74% | * | 1% |
| **Mean** | | | 48% | 12% | 24% | 13% | 5% | 27% | 38% | 58% | 5% | 28% | 6% | 8% |
| **95% CI Lower Limit** | | | 31% | 6% | 18% | 9% | 5% | 17% | 31% | 38% | 2% | 17% | 0.1% | 5% |
| **95% CI Upper Limit** | | | 65% | 17% | 30% | 17% | 6% | 37% | 44% | 79% | 7% | 39% | 11% | 11% |

Panel B2 note. Leaching fractions calculated for tampons saturated in simulated menstrual fluid. Tampons with total metals or with leachates below the method detection limit (MDL) are indicated with *. Fe, Hg, and Se are not shown since all samples were below the MDL for either the metal leachate or total metal data. CI = confidence interval.

**Supplemental Table 6.** Mean amounts of metals measured in 12 tampons representing six unique brand–product line–absorbency combinations (nanograms/gram).

| Brand | Product Line | Absorbency | As | Ba | Cd | Co | Cu | Fe | Mn | Ni | Pb | Se | Sr | V | Zn |
| --- | --- | --- | --- | --- | --- | --- | --- | --- | --- | --- | --- | --- | --- | --- | --- |
| F | 9 | Regular | 14.8 | 414 | 2.84 | 27.7 | 148 | 35739 | 559 | 539 | 63.4 | 1.89 | 617 | 70.0 | 8024 |
| G | 10 | Regular | 3.03 | 337 | 2.14 | 3.19 | 101 | 6450 | 537 | 81.1 | 31.9 | 1.46 | 834 | 39.3 | 20968 |
| I | 12 | Regular | 2.86 | 5116 | 12.6 | 57.4 | 197 | 2093 | 355 | 159 | 234 | 1.87 | 161 | 6.22 | 87882 |
| I | 12 | Super | 3.51 | 5799 | 15.3 | 47.9 | 235 | 2766 | 504 | 209 | 291 | 2.05 | 195 | 8.55 | 77473 |
| J | 14 | Regular | 2.42 | 10185 | 3.31 | 24.6 | 359 | 1885 | 46.3 | 335 | 51.5 | 2.37 | 293 | 4.61 | 17507 |
| K | 15 | Regular | 2.95 | 11500 | 11.3 | 23.5 | 252 | 1939 | 62.0 | 365 | 67.4 | 0.88 | 317 | 5.21 | 22398 |

**Note.** Cr and Hg are not reported since all samples were below the method detection limit (MDL).

**Supplemental Table 7.** Comparison of metal concentrations extracted from tampons in Jin et al. (2026) and the current study, according to extraction medium.

|  | **Jin et al. (2026)** | | **Current Study** | | | |
| --- | --- | --- | --- | --- | --- | --- |
| **Experimental Conditions** | **Acidified Water**  **pH = 5**  **at 50°C for 24 hours** | | **Simulated Vaginal Fluid**  **pH = 4.2**  **at 37 °C for 8 hours** | | **Simulated Menstrual Fluid**  **pH = 7.4**  **at 37 °C for 8 hours** | |
|  | **Median** | **Maximum** | **Median** | **Maximum** | **Median** | **Maximum** |
| **Element** | **ng/g product** | | **ng/g product** | | **ng/g product** | |
| As | 1.42 | 2.40 | 3.19 | 6.87 | 2.25 | 4.38 |
| Ba | 8.44 | 53.5 | 444 | 1517 | 130 | 417 |
| Be | <MDL | <MDL | NR | NR | NR | NR |
| Ca | 65.6 | 5922 | NR | NR | NR | NR |
| Cd | 0.159 | 0.500 | 1.52 | 7.86 | 1.40 | 6.38 |
| Co | 0.306 | 1.40 | 3.95 | 11.9 | 3.22 | 8.32 |
| Cr | 3.78 | 17.3 | 5.70 | 10.6 | 4.16 | 7.07 |
| Cu | 4.34 | 9.60 | 66.8 | 310 | 43.8 | 55.7 |
| Fe | 49.4 | 160 | 146 | 701 | <MDL | <MDL |
| Hg | <MDL | <MDL | 4.07 | 9.52 | <MDL | <MDL |
| Li | 9.43 | 19.4 | NR | NR | NR | NR |
| Mn | 7.24 | 159 | 131 | 527 | 143 | 286 |
| Mo | 5.24 | 10.8 | NR | NR | NR | NR |
| Ni | 2.44 | 4.90 | 22.1 | 117 | 59.1 | 108 |
| Pb | 1.20 | 3.50 | 4.26 | 97.0 | 4.38 | 19.7 |
| Sb | 11.6 | 120 | NR | NR | NR | NR |
| Se | <MDL | 7.80 | <MDL | <MDL | <MDL | <MDL |
| Sr | 2.08 | 464 | 67.3 | 986 | 22.3 | 1007 |
| Ti | 15.7 | 287 | NR | NR | NR | NR |
| V | 1.00 | 4.90 | 0.330 | 9.31 | 2.51 | 4.91 |
| Zn | 178 | 1540 | 4508 | 38996 | 2982 | 17483 |

**Note.** Values are medians and maxima expressed as nanograms of extracted metal per gram of product (ng/g). For each product, the extracted metal mass was divided by the product weight. Values for Jin et al. (2026) were calculated from data reported in Supplementary Tables S5 and S2.2 of that study. Extraction media and experimental conditions differed between studies: Jin et al. used acidified water of pH of 5.0, at 50°C, for 24 hours, whereas the current study used simulated vaginal (pH 4.2) and menstrual fluids (pH 7.4) at 37 °C for 8 hours. The data reported by Jin et al. did not permit calculation of the percentage of total metal content leached; therefore, percentages leached could not be compared between studies. MDL = method detection limit; NR = not reported.

**Supplemental Figure 1.** Distributions of metal amounts leached from tampons into simulated vaginal fluid (blue diamonds; pH 4.2) and simulated menstrual fluid (red circles; pH 7.4). Amounts are expressed in nanograms (ng). Grey-filled markers with blue or red outlines identify measurements below the metal-specific method detection limit (MDL) in vaginal or menstrual fluid, respectively.

**Supplemental Figure 2.** Distributions of metal amounts leached from tampons into simulated vaginal fluid (blue diamonds; pH 4.2) and simulated menstrual fluid (red circles; pH 7.4), stratified by place of purchase. Amounts are expressed in nanograms (ng). Grey-filled markers with blue or red outlines identify measurements below the metal-specific method detection limit (MDL) in vaginal or menstrual fluid, respectively. US, United States; EU, European Union; UK, United Kingdom.
